# Improving Bulk and Interfacial Lithium Transport in Garnet-Type Solid Electrolytes through Microstructure Optimization for High-Performance All-Solid-State Batteries

**DOI:** 10.1021/acsami.4c13891

**Published:** 2024-10-28

**Authors:** Young-Geun Lee, Seonghwan Hong, Bonian Pan, Xinsheng Wu, Elizabeth C. Dickey, Jay F. Whitacre

**Affiliations:** † Department of Materials Science and Engineering, Carnegie Mellon University, Pittsburgh ,Pennsylvania15213, United States of America; ‡ Scott Institute for Energy Innovation, Carnegie Mellon University, Pittsburgh, Pennsylvania15213, United States of America

**Keywords:** all-solid-state batteries, garnet-type solid electrolyte, additive chemistry, improved microstructure, suppression of dendritic behavior

## Abstract

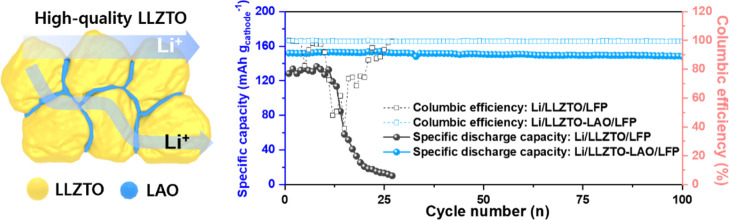

Garnet-type Li_6.4_La_3_Zr_1.4_Ta_0.6_O_7_ (LLZTO) is regarded as a highly competitive next-generation solid-state electrolyte for all-solid-state lithium batteries owing to reliable safety, a wide electrochemical operation window of 0–6 V versus Li^+^/Li, and a superior stability against Li metal. Nevertheless, insufficient interface contacts caused by pores, along with Li dendrite growth at these voids and grain boundary regions, have hindered their commercial application. Herein, we suggest a method to produce high-quality LLZTO using LiAlO_2_ (LAO) as a chemical additive that leads to an improved microstructure with larger grain size (∼25 μm), a high relative density (∼96%), lower porosity (∼3.7%), and continuous secondary phases in grain boundary regions. This improved structure results in (i) improved Li-ion conductivity and enhanced interfacial resistance between Li metal and LLZTO by a denser structure with fewer pores and (ii) suppression of Li dendrite penetration in the electrolyte by secondary phases in grain boundary regions.

## 1 Introduction

With high demand for safety, all-solid-state lithium batteries (ASSBs) have been considered as alternatives to conventional lithium-ion batteries (LIBs) because they are vulnerable to explosion hazards associated with liquid electrolytes having low flash points and high flammability.^1,2^ Solid-state electrolytes (SSEs) offer attractive merits such as improved safety and energy densities of ASSBs.^3^ In fact, SSEs can enable Li metal anodes with a high theoretical capacity of 3860 mAh g^–1^, which can provide a desirable energy density for practical applications.^4^ Among various types of SSEs, Li_7_La_3_Zr_2_O_7_ (LLZO) is a promising candidate for ASSBs due to its various advantages such as high ionic conductivity, wide electrochemical operation window (0–6 V versus Li^+^/Li), and superior chemical stability against Li metal.^5,6^

Nevertheless, the use of LLZO as SSEs for ASSBs remains hindered by several problems such as poor interfacial resistance between the Li anode and electrolyte and Li dendrite formation and growth, which is facilitated by pore phases and grain boundary regions in the microstructure.^6−8^ Indeed, voids in the microstructure can lead to a high tortuosity of Li transport, insufficient contact between LLZO and Li metal, and irregular Li-ion flux distribution.^6,9^ In addition, grain boundary regions with voids allow for the formation and growth of Li dendrites, resulting in cell short-circuiting.^10,11^ Thus, it is necessary to develop an advanced microstructure with a high density and continuous secondary phase in the grain boundary region.

Achieving both a dense structure and the desired cubic phase of LLZO typically requires a high sintering temperature exceeding 1200 °C, where lithium loss can occur, potentially resulting in poor ionic conductivity.^12−14^ Sintering additives are commonly used in LLZO to achieve a higher relative density, mitigating lithium loss by lowering the sintering temperature.^15^ After the sintering process, additives can be categorized into two groups: (i) those incorporated into the grain, potentially altering the grain ionic conductivity, and (ii) those present at grain boundaries, contributing to continued secondary phases in grain boundary regions and an altered grain boundary resistance.^16,17^ As such, various studies have devoted significant efforts to improving the LLZO microstructure by using sintering additives such as Li_4_SiO_4_, Li_3_BO_3_, and Li_3_PO_4_. Among the candidate sintering additives, LiAlO_2_ (LAO) can be a desirable sintering agent to improve the microstructure of LLZO. It can lower sintering temperature and serve as a Li-ion conducting agency in grain boundary regions, decreasing grain boundary resistance.^12,18^ In addition, secondary phases in grain boundary regions can inhibit the formation and growth of Li dendrites by reducing electronic conductivity in those areas.^11^ The high electronic conductivity at grain boundaries facilitates the release of free electrons, which combine with Li ions, reducing them to metallic Li. This promotes the formation and growth of lithium dendrites along the grain boundaries.^19−21^

Here, we explored a new tactic to simultaneously improve the microstructure of Ta-doped LLZO (LLZTO) and its electrochemical performance by introducing the effects of an LAO additive. We demonstrated the effects of LAO on the electrochemical performance parameters such as cell overpotentials and degradation behaviors. To this end, a high-quality LLZTO was designed with an improved microstructure, including a larger grain size and lower porosity. In addition, LAO additives exist at the grain boundaries after sintering and form a continuous secondary phase in grain boundary regions in the LLZTO microstructure. The improved microstructure by the multifunctional LAO additive offered the following electrochemical advantages: (i) a denser structure with a lower porosity improves bulk ionic conductivity and lowers interfacial resistance between the Li anode and the electrolyte; (ii) the larger grain size and continuous secondary phase in grain boundary regions decrease the grain boundary resistance and enhance the total ionic conductivity; and (iii) the secondary phase in grain boundary regions hinders the formation of Li dendrites at those sites and thus suppresses short-circuit of the cells.

## 2 Experimental Section

### 2.1 Preparation of LLZTO Solid Electrolyte

LLZTO (∼500 nm) was purchased from MSE Supplies LLC. For the synthesis of the LAO additive, 0.5 M aluminum nitrate (Al(NO_3_)_3_) and 0.5 M lithium nitrate (LiNO_3_) were dissolved in D.I. water and stirred for 2 h. Then, 1 M citric acid was added to the mixed solution, and the prepared solution was evaporated at 80 °C until a yellowish gel was formed. Thereafter, the sample was dried in an oven at 120 °C for 24 h. The obtained powder was calcined in a box furnace at 900 °C for 4 h. Finally, LLZTO and 1 wt % LAO were mixed by a vortex mixer, pressed into a pellet at 78.5 MPa, and sintered at 1100 °C for 10 h with the mother powder. After sintering, the LLZTO-LAO pellets were polished with 600- and 800-grit sandpaper using isopropyl alcohol to clean the surface.

### 2.2 Material Characterization

To investigate the structural properties of prepared samples, we performed X-ray diffraction (XRD) in the 2θ range of 10–60° using Cu Kα radiation. The relative density of prepared pellets was measured by geometric measurement, the Archimedes method, and XRD results. The porosity of the sintered LLZTO pellets was further characterized via X-ray microcomputed tomography (X-ray microCT, Zeiss Group) with a minimum voxel size (resolution) of ∼1.6 μm, a voltage of 140 kV, and a current of 71 mA. The resulting X-ray CT image was processed using ORS Dragonfly software (Comet Technologies Canada Inc.), focusing on a central region measuring 1.5 mm × 1.5 mm × 0.4 mm. The microstructures and elemental distributions were investigated using field-emission scanning electron microscopy (FE-SEM) with energy-dispersive spectrometry (EDS).

### 2.3 Electrochemical Measurements

To investigate the ionic conductivity of the LLZTO solid-electrolyte pellets, a Li-ion blocking electrode was employed by sputtering Au (Au) onto both sides of the electrolyte. Electrochemical impedance spectroscopy (EIS) measurements were performed by Solatron 1260 in a frequency range from 5 MHz to 100 Hz at various temperatures of 0 to 40 °C. To measure the interfacial resistance between Li and LLZTO electrolyte, Li-symmetric cells were fabricated. Li-metal (8 mm diameter) was attached to both surfaces of the electrolyte by applying 3.0 MPa, heating on a hot plate at 200 °C for 30 min, and sealing into a 2032-type coin cell. Using this cell, we conducted EIS measurements over the frequency range from 1 MHz to 100 Hz at room temperature. Galvanostatic Li-plating/stripping tests of the symmetric cells with a current density of 0.1 mA cm^–2^ were performed up to 300 h with 0.5 h duration of each cycling step. The critical current density (CCD) was also investigated by galvanostatic cycling at current densities ranging from 0.05 to 0.7 mA cm^–2^.

In addition, electrochemical measurements were performed using a full-cell system consisting of Li metal as the anode, LLZTO-LAO as an electrolyte, and lithium phosphate (LFP) as a cathode. The cathode was prepared by mixing LFP with poly(vinylidene fluoride) (PVDF) and Super-P carbon black in a weight ratio of 8:1:1. To improve the interfacial contact between the electrolyte and cathode, 1 μL of liquid electrolyte (1 M LiPF_6_ EC/DMC) was added on the cathode. The rate capability of this cell was measured at various rates of C/20, C/10, C/5, C/3, and C/2, and a cycling stability test was carried out at a rate of C/10 up to 100 cycles.

## 3 Results and Discussion

**Figure 1:**
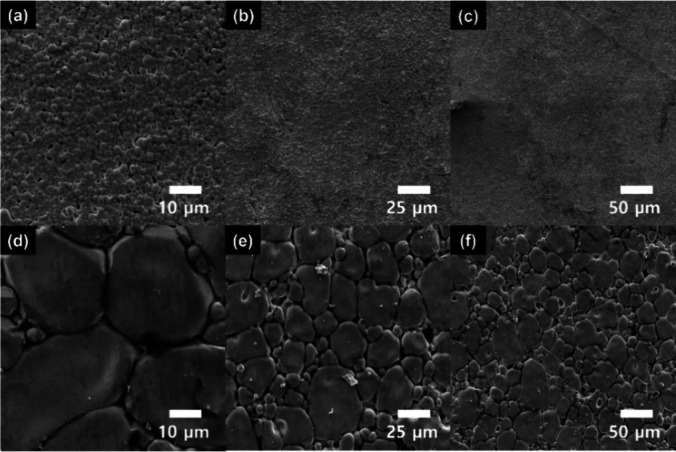
Top-view SEM images of (a–c) LLZTO and (d–f) LLZTO-LAO.

Obtaining a high-quality LLZTO pellet with an improved microstructure is crucial to its use as an SSE for high-performance ASSBs. Therefore, in this work, the LLZTO electrolyte with an enhanced microstructure is fabricated by introducing a multifunctional LAO additive into LLZTO. To elucidate the morphologies and microstructures of prepared samples, the top-view SEM images are obtained, as shown in Figure 1a–f. While the LLZTO (Figure 1a–c) exhibits a smaller average grain size (<1 μm) with numerous pores in the microstructure, the LLZTO-LAO (Figure 1d–f) demonstrates a notably improved microstructure with a larger average grain size (∼25 μm) and a denser structure. In addition, the cross-sectional SEM images in Figure S1 reveal the interior microstructure of prepared pellets, wherein LLZO-LAO shows an improved microstructure with larger grain sizes and lower porosity than that of LLZTO. These enhanced properties of the LLZTO-LAO are attributed to the LAO additive.

**Figure 2:**
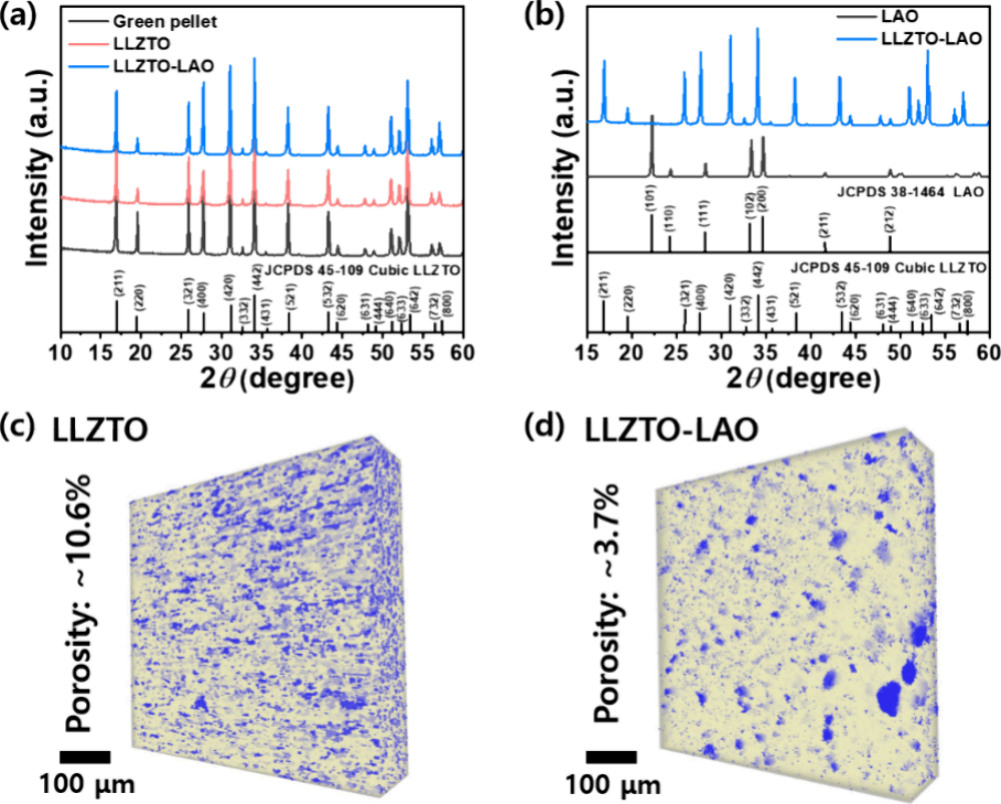
Crystal phases and porosities of LLZTO and LLZTO-LAO: (a, b) XRD results and (c, d) X-ray microCT results.

To investigate the crystal structures of prepared pellets, XRD measurements are conducted as shown in Figure 2. Interestingly, regardless of the preparation method, all the LLZTO pellets demonstrate a cubic phase, but no indication of the LAO phase is evident, which is further confirmed by the XRD result with a semilog scale in Figure S2. Based on the XRD data, geometric measurement, and Archimedes method, the relative densities of green pellet without sintering additive and LLZTO are calculated to be ∼65 and ∼88%, respectively. For LLZTO-LAO, the theoretical density (∼5.4 g cm^–3^) and relative density (∼96%) are calculated by the rule of mixtures, incorporating the density of the LAO additive in Table S1. In addition, the porosity of various samples was investigated by X-ray microCT images with a resolution of 2.0 μm. As shown in Figure 2c,d, the porosities of LLZTO and LLZTO-LAO were found to be ∼10.6 and ∼3.7%, respectively, which correlate with the results of the SEM images and relative density.

**Figure 3:**
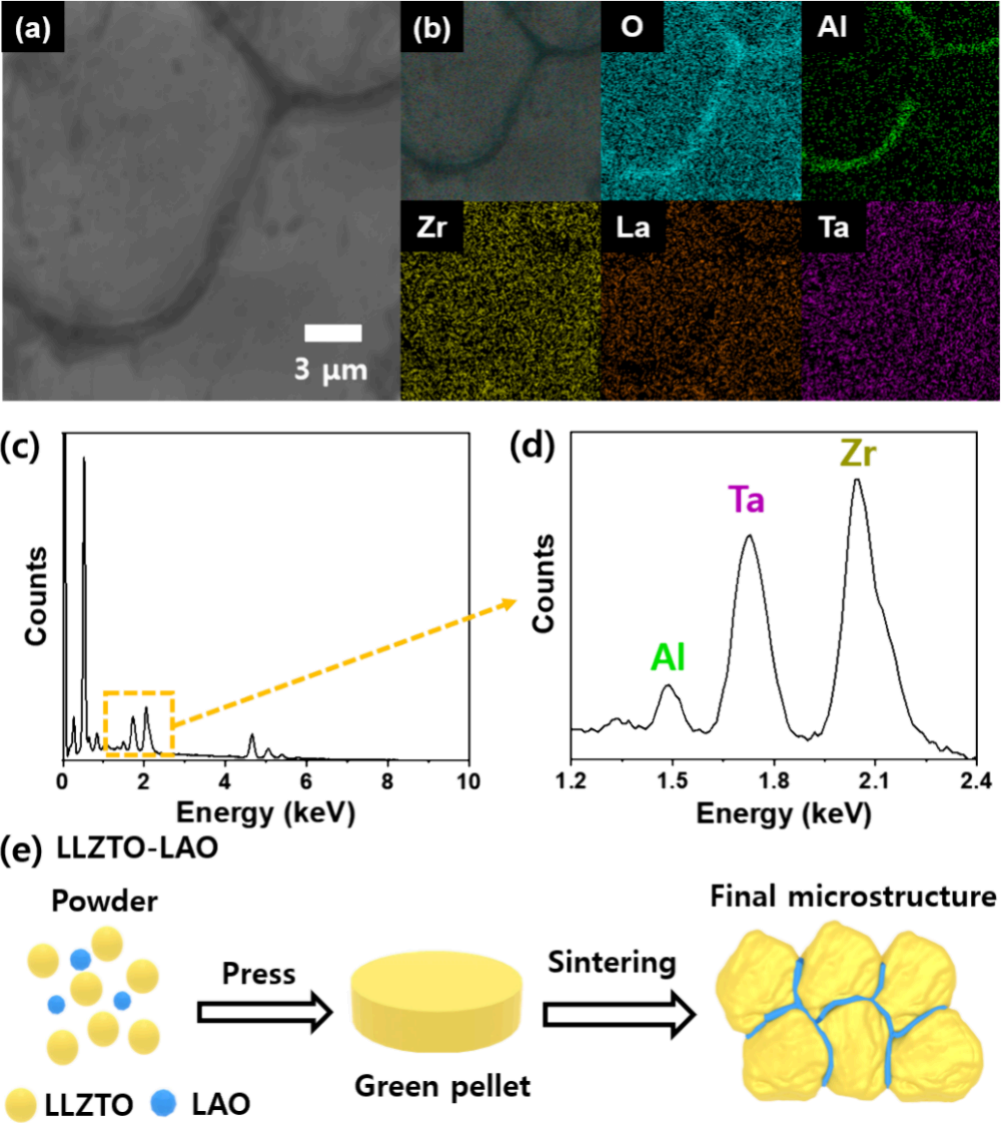
Morphology and elemental distribution of LLZTO-LAO: (a) SEM secondary electron (SE) images with (b) EDS maps and (c, d) EDS spectra. (e) Schematic showing the fabrication of LLZTO-LAO solid-state electrolytes (SSEs).

The presence of LAO in the microstructure of LLZTO-LAO was not conclusively demonstrated by the SEM and XRD results. Hence, the elemental distribution of LLZTO-LAO was further investigated by SEM-EDS analysis, as shown in Figure 3. The SEM-EDS mapping images in Figure 3b reveal that the elements oxygen (O), zirconium (Zr), lanthanum (La), and tantalum (Ta) are uniformly distributed within the grain, whereas aluminum (Al) is highly segregated along the grain boundaries. Moreover, the EDS spectra from the grain boundary region (Figure 3c,d) confirmed the presence of Al elements in the LLZTO-LAO, suggesting that Al elements are present in the grain boundary. To further demonstrate the existence of LAO in the grain boundary, the EDS-mapping was conducted for the grain region, as shown in Figure S3. In these images, the detection of Al elements within the grain is evident, albeit with lower intensity. This observation further supports the notion that LAO phases primarily precipitate in the grain boundary rather than being fully incorporated into the grain during the sintering process. Thus, we successfully synthesized the high-quality LLZTO pellet with an enhanced microstructure with a lower porosity and secondary phase in the grain boundary region using LAO additives, as shown in Figure 3e.

**Figure 4:**
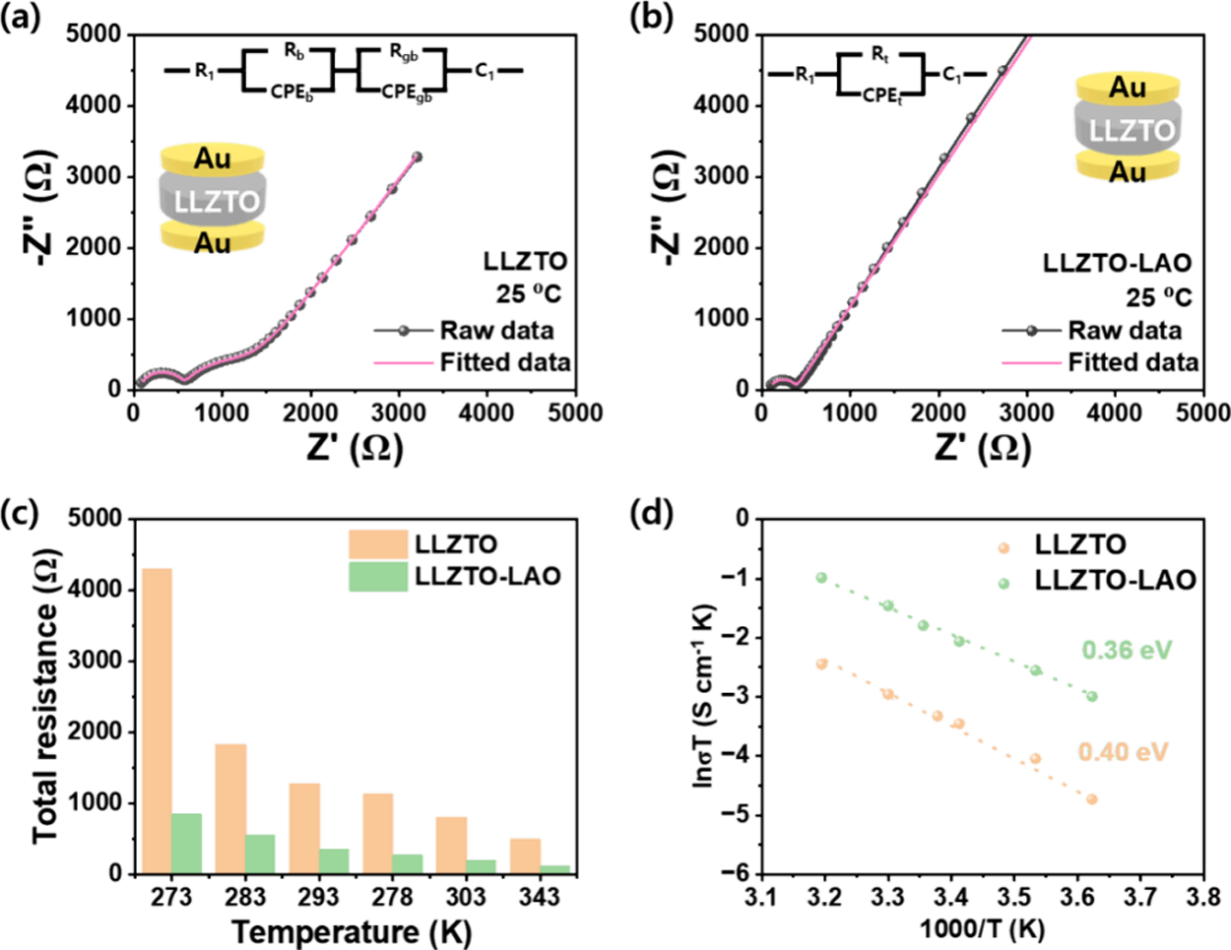
(a, b) Nyquist plots of Li-ion blocking electrodes with an equivalent circuit model. (c) Total (bulk and grain boundary) resistance. (d) Arrhenius plots with total activation energy of LLZTO and LLZTO-LAO.

To clarify the role of an improved microstructure on the electrochemical performance of LLZTO-LAO, the ionic conductivity and activation energy for lithium transport were investigated via EIS in a temperature range of 0 to 40 °C using Li-ion blocking electrodes of sputtered Au on both sides of the pellets. In Figure 4a, the Nyquist plots for LLZTO depicted two distinct semicircle regions, reflecting the bulk resistance at high frequencies and the grain boundary resistance at lower frequencies, respectively. To model this impedance behavior, the equivalent circuit model of (*R*_c_)(*R*_b_*C*_b_)(*R*_gb_*C*_gb_)(*C*_be_) is employed, where b, gb, and be represent the bulk, grain boundary, and blocking electrode, respectively. The Nyquist plots for LLZTO with various temperatures ranging from 0 to 40 °C are also presented in Figure S4. On the other hand, the Nyquist plot of LLZTO-LAO reveals an incomplete high-frequency semicircle, indicative of the overlap between bulk and grain boundary resistance, as depicted in Figures 4b and S5. To accurately represent this impedance behavior, the equivalent circuit model of (*R*_c_)(*R*_t_*C*_t_)(*C*_be_) is employed for fitting, where t denotes the total resistance encompassing both bulk and grain boundary contributions. In Figure 4c, the total resistance is depicted across different temperature ranges, providing insight into the temperature-dependent behavior. Additionally, Table S2 presents the bulk, grain boundary, and total resistance values alongside the Li-ion conductivity at room temperature. The decreased total resistance in the LLZTO-LAO is attributed to an enhanced relative density that can decrease tortuosity. Meanwhile, the reduction in grain boundary resistance may result from grain growth. Additionally, the presence of LAO within these boundaries serves as a conducive medium for lithium-ion migration, further facilitating ion transport in the area.^18,22,23^ Based on the temperature-dependent resistances, the total activation energy (bulk and grain boundary) is calculated from the Arrhenius behavior, as shown in Figure 4d.^24,25^ The LLZTO-LAO shows a decreased activation energy of 0.36 eV compared to LLZTO (0.40 eV), which is attributed to an improved microstructure with ionic conductive secondary phases in grain boundary regions. For comparison, SEM images and EIS results for the LLZTO with varying LAO ratios (0.5 and 2.0 wt %) are also obtained in Figure S6 and Table S3. Thus, it is expected that LLZTO-LAO will display improved energy storage performance.

**Figure 5:**
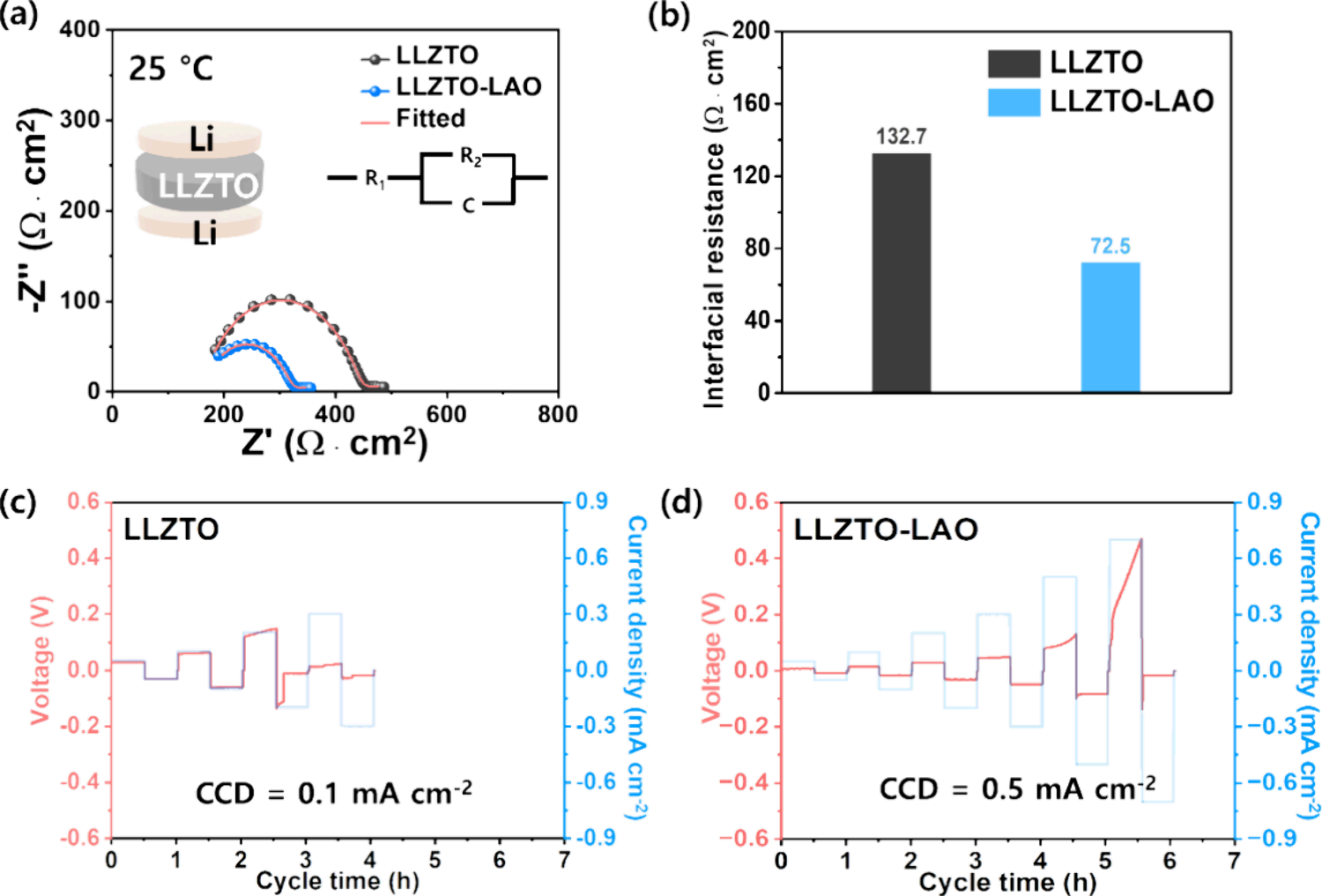
(a) Nyquist plots of Li-ion blocking electrodes with equivalent circuit model. (b) Interfacial resistances. (c, d) Critical current density results at various current densities of 0.05 to 0.7 mA cm^–2^.

Generally, interfacial resistance between the Li anode and LLZTO electrolyte has a significant impact on electrochemical performance, including overpotential and CCD. Hence, to investigate the interface resistance and demonstrate its effect on the electrochemical performance, EIS measurements were conducted using a Li–Li symmetric cell, as shown in Figure 5a. In the Nyquist plots, semicircles indicate the interface resistance without diffusion slopes, confirming the general behaviors of Li–Li symmetric cells.^26,27^ In particular, the interfacial resistance was calculated to be 132.7 and 72.5 Ω cm^2^ for LLZTO and LLZTO-LAO, respectively, as shown in Figure 5b. In addition, to measure the CCD, the galvanostatic Li plating/stripping test was performed at various current densities from 0.05 to 0.7 mA cm^–2^ with a 0.5 h duration of each cycling step in Figure 5c,d. While LLZTO (Figure 5c) delivers a CCD of 0.1 mA cm^–2^, a higher CCD of 0.5 mA cm^–2^ is demonstrated within the symmetric cell of LLZTO-LAO (Figure 5d). In addition, the LLZTO-LAO showed lower overpotentials than the LLZTO at the various current densities. The CCD and overpotentials of the LLZTO-LAO can be attributed to the use of LAO sintering additive that leads to an improved microstructure with a reduced porosity, which results in an enhanced Li-ion transport and good interfacial contact between the electrolyte and Li metal. In other words, a higher porosity in the LLZTO cell leads to nonuniform Li-ion flux, higher tortuosity, increased interfacial resistances, and thus higher overpotentials. Thus, these results imply that the enhanced microstructure of LLZTO-LAO can mitigate the risk of short-circuiting to a certain degree.

**Figure 6:**
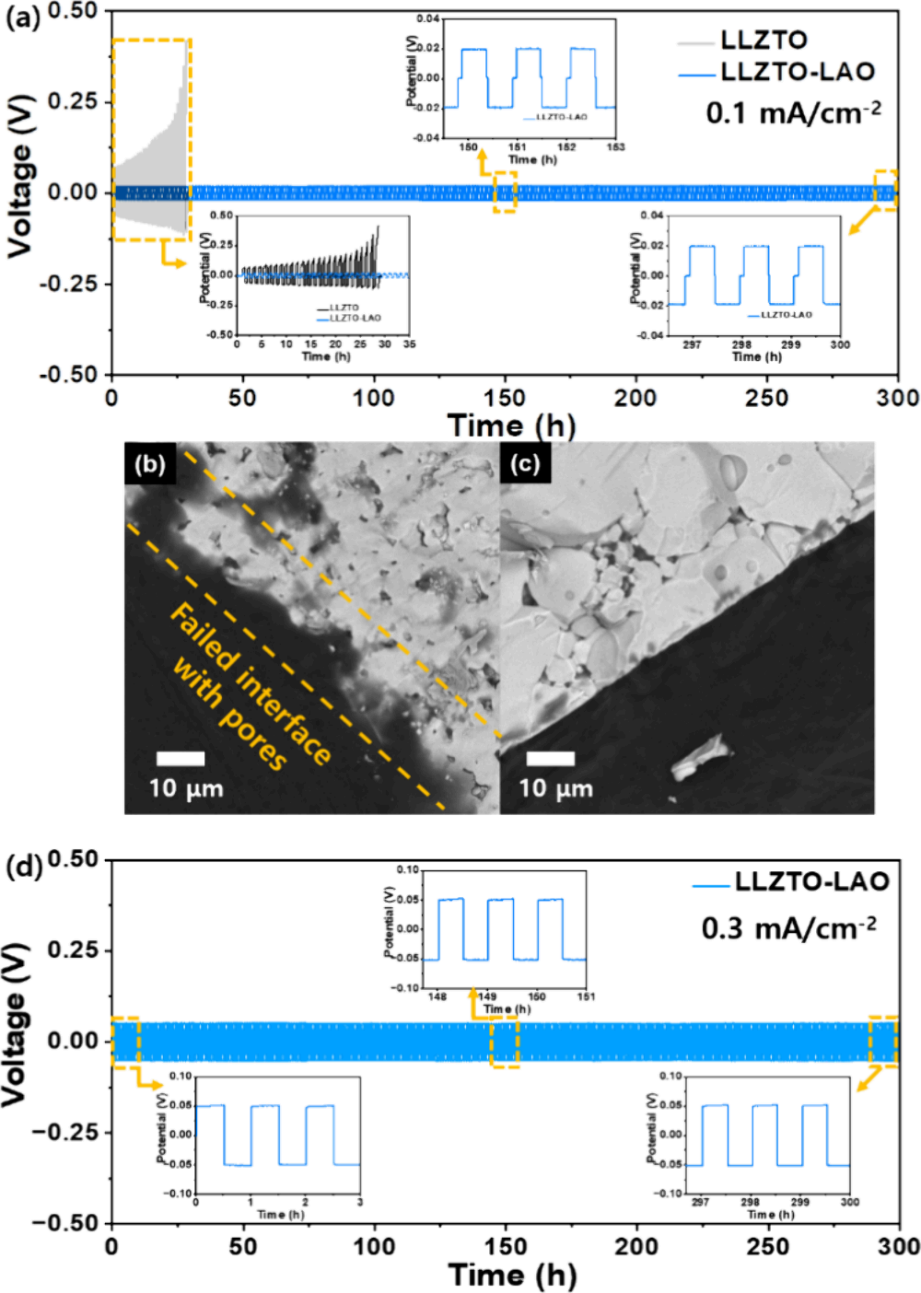
Li-symmetric cell performance and cross-sectional SEM backscattered electron (BSE) images using LLZTO and LLZTO-LAO: (a) Long-term stability at a current density of 0.1 mA cm^–2^. Cross-sectional BSE images of (b) LLZTO and (c) LLZTO-LAO after cycling. (d) Long-term stability at a current density of 0.3 mA cm^–2^.

To further investigate the effect of the improved microstructure of LLZTO-LAO on the electrochemical performance, a long-term Li plating/stripping test was conducted at a current density of 0.1 mA cm^–2^ with a 0.5 h duration of each cycling step up to 300 cycles, as shown in Figure 6a. As expected from the EIS and CCD results, the LLZTO-LAO exhibits similar overpotentials (∼20 mV) without short-circuit after 300 cycles. On the contrary, the overpotentials of the LLZTO cell gradually increased, and short-circuit occurred after 28 cycles. The short-circuit problem in LLZTO might be attributed to uneven Li-ion transport at the interface, as well as the formation and propagation of lithium dendrite in the pore phases and grain boundary regions. The interfacial structure of prepared cells was characterized by using cross-sectional SEM images using backscattered electrons (BSEs) after long-term cycling, as shown in Figure 6b,c. Here, the LLZTO cell shows an uneven and failed interface between the electrolyte and Li metal, leading to considerable degradation of the interface and Li-ion flux during repeated Li-stripping/deposition and thus causing the short-circuit of the cell. In addition, numerous pores and voids in the LLZTO can serve as sites for the formation and propagation of Li-dendrite. On the contrary, the LLZTO-LAO cell reveals a uniform interface without the formation of defects at both the surface and the inside of the electrolyte due to the large grain size and highly compacted microstructure. In addition, the long-term stability test for the Li-symmetric cell with LLZTO-LAO at a higher current density of 0.3 mA cm^–2^ was conducted, as shown in Figure 6d. Therefore, these results demonstrated that the improved microstructure of LLZTO-LAO is effective in the interfacial resistance, suppression of Li-dendrite formation, and electrochemical performance.

**Figure 7:**
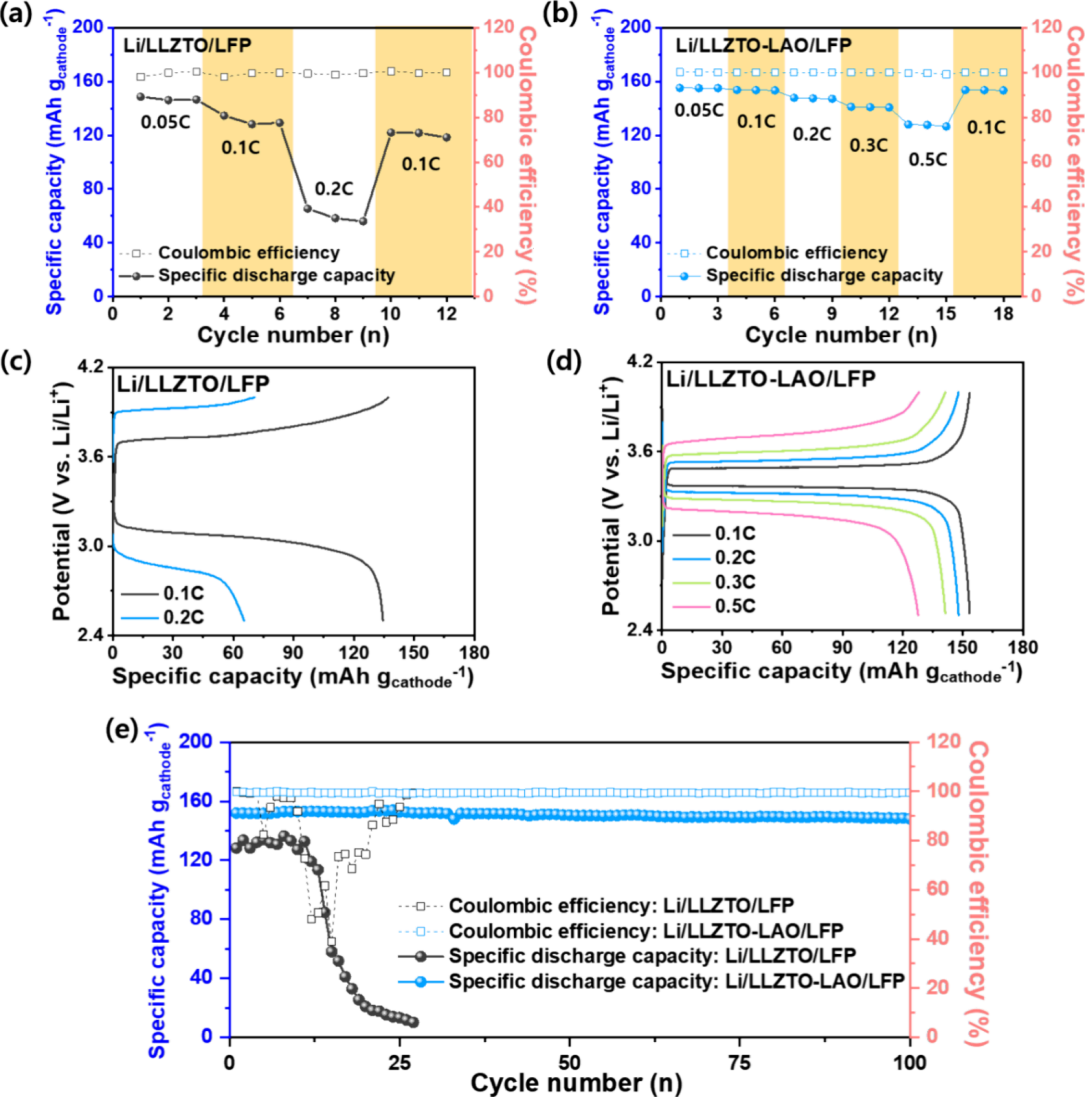
Hybrid full-cell performance: Rate performances of (a) Li/LLZTO/LFP and (b) Li/LLZTO-LAO/LFP at various rates of 0.05, 0.1, 0.2, 0.3, and 0.5 C. Charge/discharge curves of (c) Li/LLZTO/LFP and (d) Li/LLZTO-LAO/LFP according to the rate performance. (e) Cycling stability at the rate of 0.1 C after the rate performance.

To demonstrate the feasibility of our tactics for ASSB electrolyte, the energy storage performance was conducted using the hybrid full-cell system composed of an LLZTO or LLZTO-LAO electrolyte, Li metal anode, and LiFePO_4_ (LFP) cathode. Figure 7a,b shows the rate performance of prepared cells at room temperature and various current rates of 0.05 to 0.5 C. Notably, the rate performance of the full cell with LLZTO (Figure 7a) was evaluated only up to a current rate of 0.2 C owing to significant overpotential observed at the higher current rates originating from the poor interfacial resistance. In contrast, Li/LLZTO-LAO/LFP exhibits an improved rate performance with a retention rate of ∼99% (from 153.2 to 151.7 mAh g^–1^). To investigate the overpotentials of prepared cells, charge/discharge curves were also prepared as shown in Figures 7c,d, and S7. At the current rate of 0.1 C, Li/LLZTO-LAO/LFP displays an improved voltage polarization of (0.124 V) than Li/LLZTO-LAO/LFP (0.729 V), corresponding to the Li symmetric cell results. Also, it suggests an excellent electrochemical reaction and lower resistance of the LLZTO-LAO cell. Figure 7e exhibits the cycling stability performance of prepared cells, and the LLZTO delivers poor cycling stability and Coulombic efficiency after a few cycles, which might be Li dendrite formation and propagation in the pore phases and grain boundary. As shown in Figure S8, the charge and discharge profiles show the dendritic behaviors in the LLZTO, demonstrating that Li dendrite formation and propagation can occur in the pore phases and grain boundary regions.^28^ On the contrary, the Li/LLZTO-LAO/LFP demonstrated a remarkable cycling stability of ∼96.8% after 100 cycles and dendrite-free behavior with excellent Coulombic efficiency, caused by the improved microstructure with an excellent interface between Li and electrolyte, reduced pore phases, and a continued secondary phase in grain boundary regions.

## 4 Conclusions

In summary, this work presented a design of a high-quality LLZTO for improved performance of ASSBs by exploiting multifunctional LAO sintering additives that cause an improved microstructure with a denser structure, larger grain size, and continuous secondary phases in grain boundary regions after the sintering process. Particularly, the additive effect of LLZTO electrolytes on the electrochemical performance of the ASSBs was demonstrated. First, the LAO additive improved the relative density of LLZTO and acted as an ion-conductive secondary phase in the grain boundary, thus enhancing the total ionic conductivity. Second, a denser structure of LLZTO-LAO improved the interfacial resistance between the Li metal and electrolyte, resulting in an enhanced CCD and rate performance. Lastly, lower porosity and grain boundary regions filled with secondary phases hindered the formation and propagation of undesirable and irreversible Li dendrites in the electrolyte, thus improving the cycling stability. This work has demonstrated the additive chemistry of SSEs and provides insights into the fabrication of all-solid-state batteries.

## Supporting Information Available

Lattice parameters, relative density, resistance, and ion-conductivity of LLZTO and LLZTO-LAO; cross-sectional SEM images; SEM-EDS with the interior grain regions; temperature-dependent EIS results; and charge/discharge curves with rate-performance and long-term stability results (PDF)
