# Supplementary material for: Improving Bulk and Interfacial Lithium Transport in Garnet-Type Solid Electrolytes through Microstructure Optimization for High-Performance All-Solid-State Batteries
Source: ACS Appl Mater Interfaces. 2024 Oct 28;16(44):60340–7. doi: 10.1021/acsami.4c13891 (PMC11551947; doi:10.1021/acsami.4c13891)
Supplement: Supplementary file 1 — am4c13891_si_001.pdf [file am4c13891_si_001.pdf]

Supporting Information

Improving Bulk and Interfacial Lithium Transport in Garnet-Type Solid Electrolytes through Microstructure Optimization for High-Performance All-Solid-State Batteries

Young-Geun Lee[a], Seonghwan Hong[a], Bonian Pan[a], Xinsheng Wu[a], Elizabeth C. Dickey[a*],

Jay F. Whitacre[a,b**]

[a]Department of Materials Science and Engineering, Carnegie Mellon University, Pittsburgh, 15213, United States of America

[b]Scott Institute for Energy Innovation, Carnegie Mellon University, Pittsburgh, 15213, United States of America

Corresponding author.

E-mail address:

ecdickey@andrew.cmu.edu (Elizabeth C. Dickey)

Whitacre@andrew.cmu.edu (Jay F. Whitacre)

	Lattice parameter (nm)		Density (g cm^{-3})
	a	c	
**LAO**	**0.513**	**0.625**	**2.60**
**LLZTO-LAO**	**1.293**		**5.46**

Table S1 Lattice parameters and density of LAO and LLZTO-LAO calculated by the XRD results

At 25°C	Bulk resistance (Ω)	Grain boundary resistance (Ω)	Total resistance (Ω)	Total ion conductivity (mS cm^{-1})	Activation energy (eV)
LLZTO	469.29	669.64	1138.93	0.12	0.40
LLZTO-LAO			278.54	0.56	0.36

Table S2 Bulk resistance, grain boundary resistance, total resistance, ionic conductivity, and activation energy of LLZTO and LLZTO-LAO at room temperature

At 25°C	Relative density	Total resistance (Ω)	Total ion conductivity (mS cm^{-1})
LLZTO-0.5LAO	~91%	725.18	0.23
LLZTO-2.0LAO	~97%	433.13	0.40

Table S3 Relative density, total resistance, and ion conductivity of LLZTO with varying LAO weight ratios of 0.5 wt% (LLZTO-0.5LAO) and 2.0 wt% (LLZTO-2.0LAO)

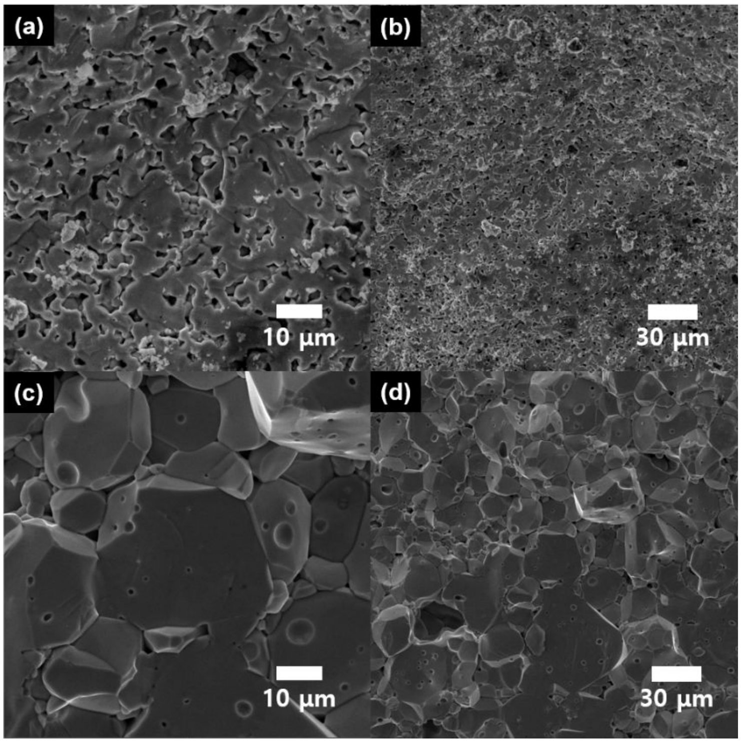

Figure S1. Cross-sectional SEM SE images of (a,b) LLZTO and (c.d) LLZTO-LAO

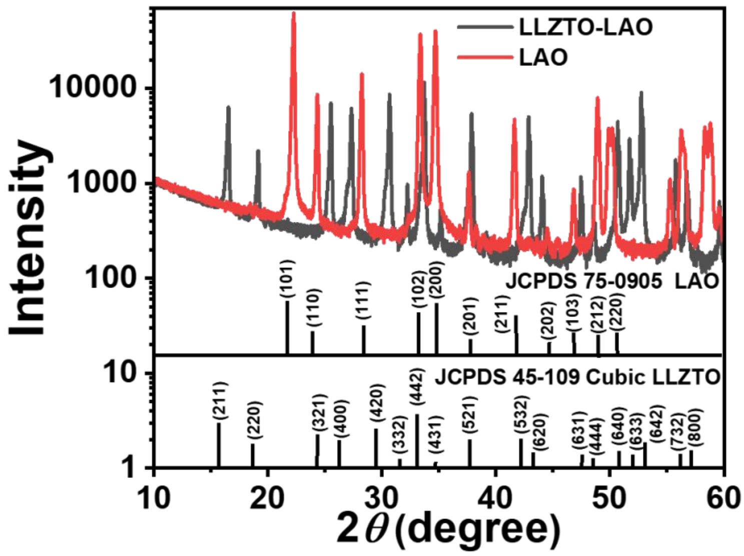

Figure S2. XRD result with a semi-log scale including LLZTO-LAO and LAO

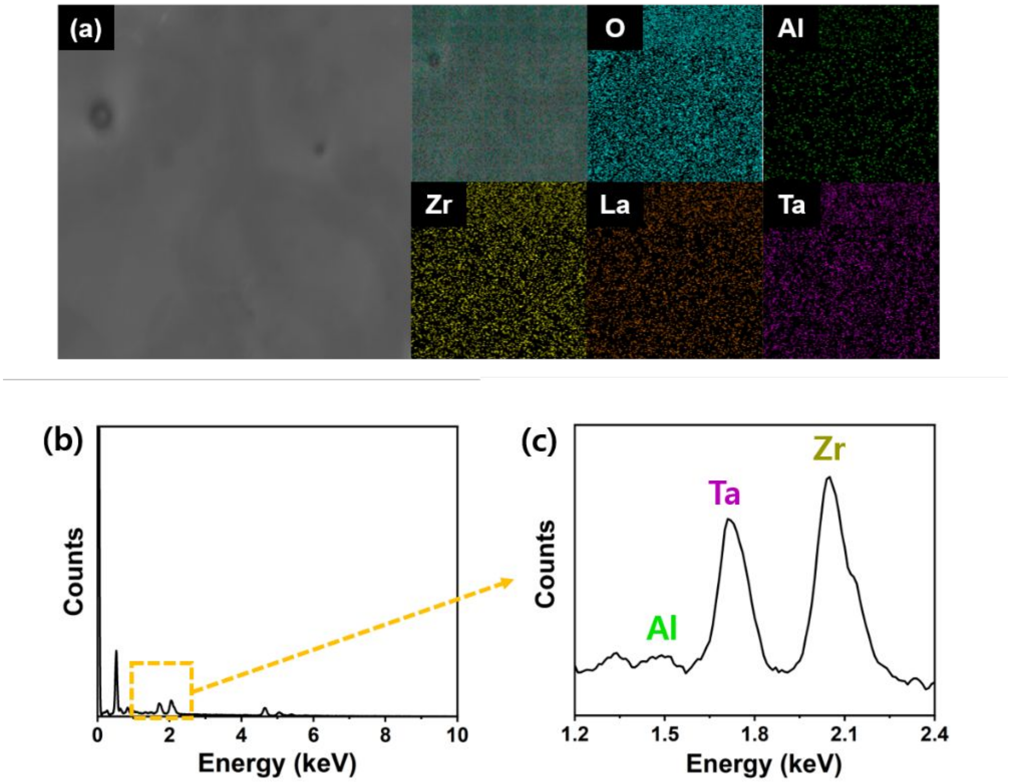

Figure S3. SEM-EDS results: (a) mapping images and (b,c) spectrum from a grain-interior region in LLZTO-LAO.

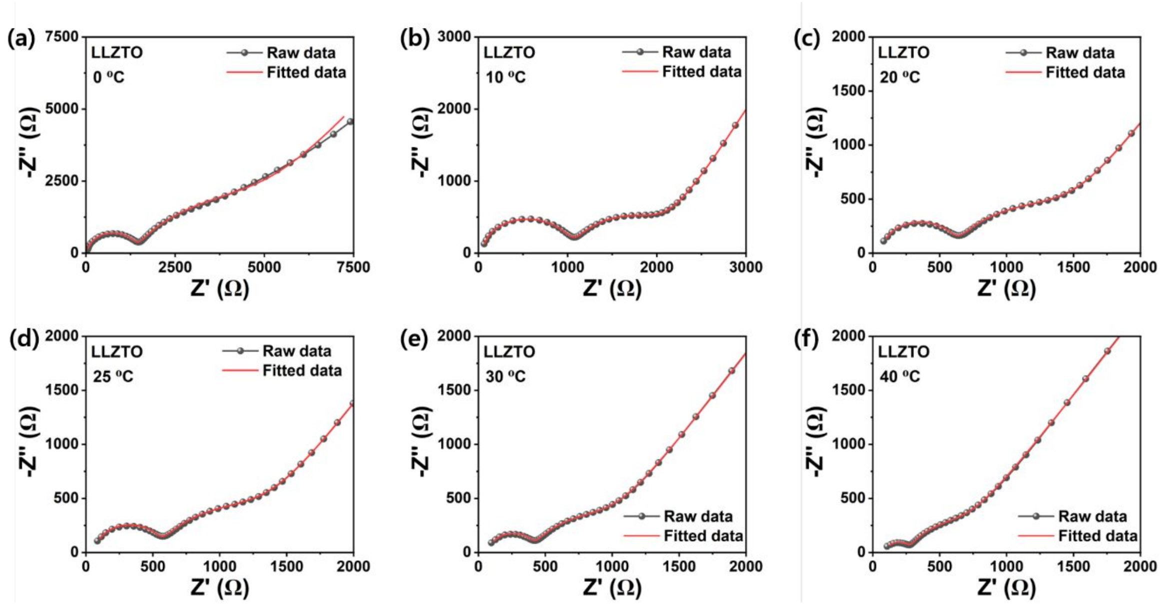

Figure S4. EIS results using Au-Au blocking electrode for LLZTO at various temperatures of of (a) 0 °C, (b) 10 °C, (c) 20 °C, (d) 25 °C, (e) 30 °C, and (f) 40 °C.

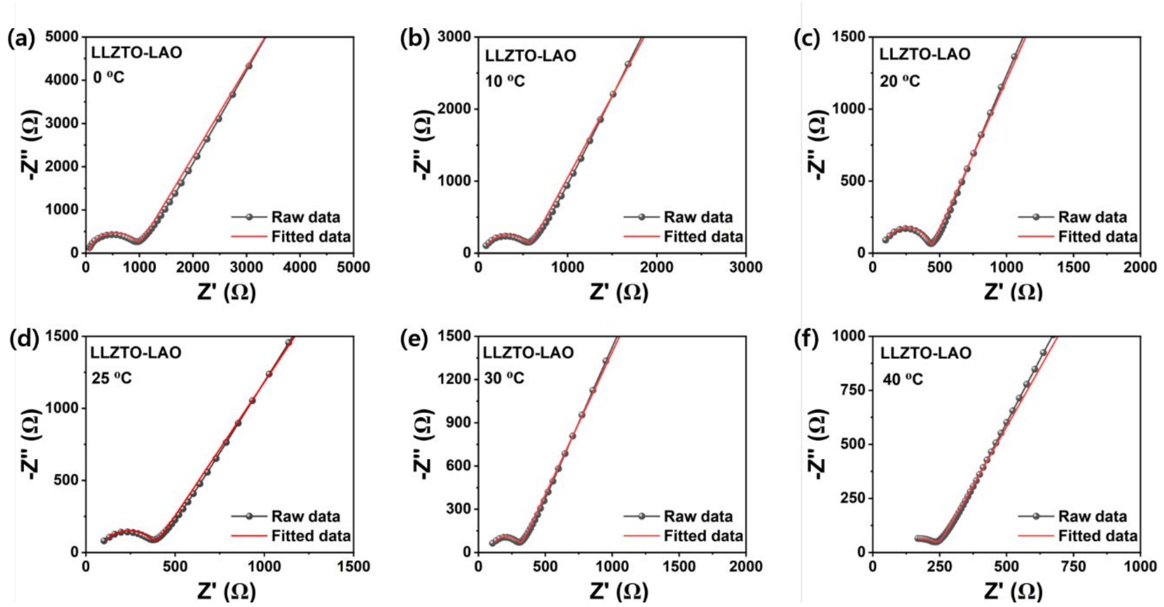

Figure S5. EIS results using Au-Au blocking electrode for LLZTO-LAO at various temperatures of (a) 0 °C, (b) 10 °C, (c) 20 °C, (d) 25 °C, (e) 30 °C, and (f) 40 °C.

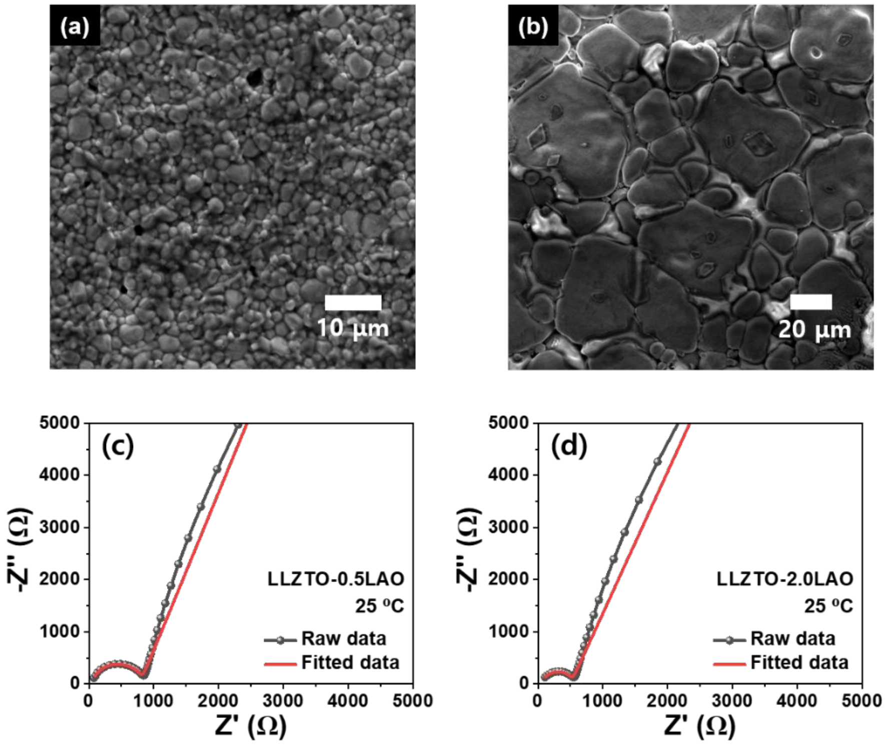

Figure S6. (a,b) SEM images and (c,d) EIS results using Au-Au blocking electrode for LLZTO with varying LAO weight ratios of 0.5 wt% (LLZTO-0.5LAO) and 2.0 wt% (LLZTO-2.0LAO) at room temperature.

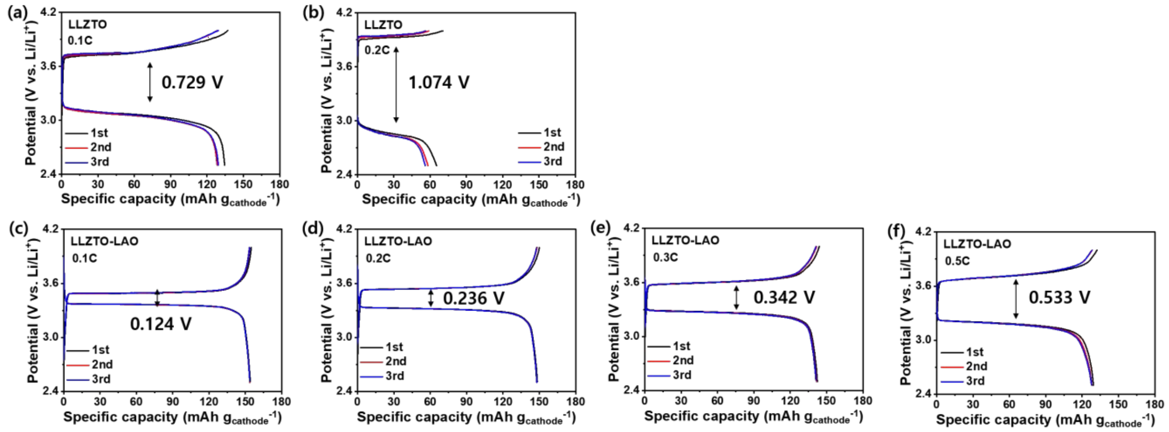

Figure S7. Charge/discharge curves within rate-performance results of (a,b) LLZTO and (c-f) LLZTO-LAO at various current densities.

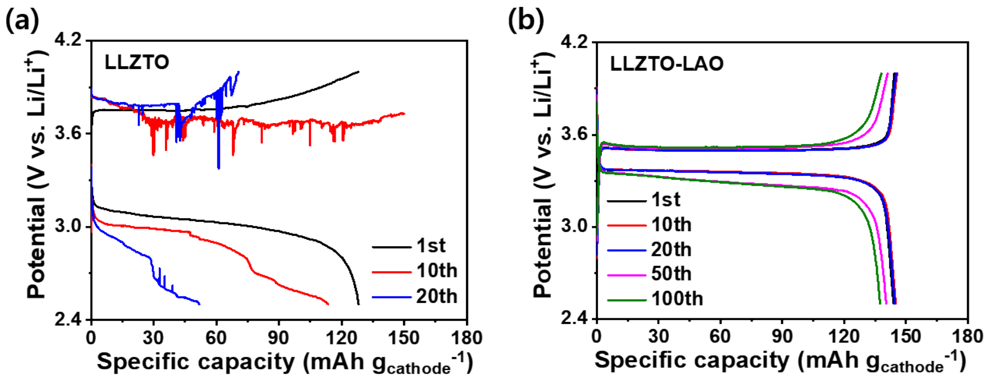

Figure S8. Charge/discharge curves within cycling stability results of (a) LLZTO and (b) LLZTO-LAO at a current density of 0.1C.
